# Engagement in Medication Communication During Transitions of Care for Rural Aged Care Residents and Family Caregivers: A Qualitative Study

**DOI:** 10.1111/jocn.70047

**Published:** 2025-07-25

**Authors:** Alison Dowling, Elizabeth Manias

**Affiliations:** ^1^ School of Nursing and Midwifery Monash University Clayton Victoria Australia

**Keywords:** aged care, family caregivers, communication, medication therapy management, residents, rural, transitions of care

## Abstract

**Aim:**

To explore how residents and caregivers experience engagement in medication communication during transitions of care.

**Design:**

Qualitative exploratory study.

**Methods:**

Nine residents and seven family caregivers from two rural aged care homes participated in semi‐structured interviews between June and July 2024. Engagement in medication communication was assessed using the Patient and Family Engagement Framework. The COREQ checklist guided reporting of the study.

**Results:**

The study identified two main themes: (1) Medication communication during transitions into healthcare services; (2) Influences shaping residents' and caregivers' engagement in medication communication. Participants primarily experienced passive consultation about medications, mainly due to a lack of proactive engagement from healthcare providers, with both intrinsic and external factors significantly hindering their involvement in medication communication.

**Conclusion:**

Participants had minimal engagement in medication communication during transitions, receiving mainly reactive, one‐way information from providers. Improved communication strategies and greater involvement of residents and caregivers are needed to enhance medication safety and continuity of care in rural settings.

**Relevance to Clinical Practice:**

This study provides insights into medication communication engagement among rural aged care residents and their family caregivers. By applying the Patient and Family Engagement Framework, the findings highlight the need for proactive, clear and inclusive communication strategies to enhance medication safety and continuity of care. Improving engagement in medication discussions can support shared decision‐making, reduce misunderstandings and improve transitions of care in rural aged care settings.

**Reporting Method:**

The study followed COREQ guidelines.

**Patient or Public Contribution:**

Residents and family caregivers participated through interviews.

**Summary**

- This study offers new insights into engagement about medication communication during transitions of care for rural aged care residents and caregivers, filling a gap in global healthcare research. It advocates for inclusive communication models that improve shared decision‐making, particularly in rural and resource‐limited settings, contributing to the global clinical community.
- The study highlights the importance of effective healthcare communication in ensuring medication safety and continuity of care, identifying barriers that affect medication engagement during transitions for rural aged care residents and their family caregivers.
- It provides valuable insights into the need for structured, proactive communication strategies, such as training for healthcare providers and the use of communication tools, to improve engagement and decision‐making among rural aged care residents and their family caregivers.

## 1 Introduction

Australia's aging population presents challenges as more individuals remain at home longer, leading to residential aged care homes (RACHs) caring for older residents with complex medical needs (Australian Institute of Health and Welfare 2025; Elliott and Booth 2014). These residents, often with multiple health conditions, are frequently prescribed numerous medications, increasing the risk of medication errors (Page et al. 2023; Wabe et al. 2024) and adverse drug events (Ye et al. 2022), often resulting in hospitalisations (Barber et al. 2009). RACH residents undergo frequent transitions of care to hospitals, where medication errors occur due to communication breakdowns during handovers, admissions and discharges involving healthcare providers, residents and family caregivers (Chhabra et al. 2013; Coleman et al. 2004). Medication communication challenges in rural RACHs are globally relevant, as ageing populations worldwide face similar healthcare demands (Díez et al. 2022; Xu et al. 2021; Özkaytan et al. 2024). Effective communication during transitions of care is a global priority (World Health Organization 2016). This study provides insights into the experiences of rural Australian RACH residents and their family caregivers, contributing to global knowledge and informing policies to enhance medication safety and care continuity.

## 2 Background

Residential aged care homes (RACHs) in Australia care for older adults unable to live independently, with over half requiring high‐level support for cognitive, behavioural and daily living challenges (Australian Government Department of Health and Aged Care 2019). These complex needs lead to frequent transitions between healthcare settings, increasing risks, particularly in medication management (Redmond et al. 2018). Residents often experience five to seven medication changes during hospitalisation, which are poorly communicated to residents and family caregivers, limiting engagement and increasing error risks (Elliott and Booth 2014; Redmond et al. 2018).

Communication breakdowns contribute to medication harm, with medication errors accounting for 20% of global medical errors and being a leading preventable cause of harm (World Health Organisation 2025). In Australia, medication errors contribute to 5%–10% of hospital incidents and 2%–3% of admissions, with RACHs particularly affected (Khalil and Roughead 2017). Errors account for 9% of adverse incidents in RACHs, while international studies estimate error rates of 16%–27% during hospital‐to‐RACH transitions, with 17% of residents experiencing adverse drug events post‐discharge (Kapoor et al. 2019; St Clair et al. 2022).

Research on RACH residents' and caregivers' engagement in medication communication during transitions is limited (Dowling et al. 2024). Medication communication, the exchange of information about medicines between healthcare providers, patients and caregivers, is essential for safe and effective use (Manias 2010). This gap is more pronounced in rural RACHs, where residents face challenges like healthcare access, provider shortages and geographic isolation (Dowling et al. 2024; Piper et al. 2018). Most studies have focused on older adults transitioning between home and hospital in urban settings, with limited research on RACH residents' experiences (Manias et al. 2019, 2020; Ozavci et al. 2021, 2022; Tobiano et al. 2019). A systematic review (Manias et al. 2020) explored the engagement of non‐rural RACH residents and caregivers in medication management, identifying key barriers and enablers. Other studies have examined medication management and shared decision‐making in metropolitan RACHs, consistently highlighting fragmented communication that does not centre on residents or caregivers (Dowling et al. 2024; Manias et al. 2020; Sawan et al. 2022).

Timely, accurate and person‐centered communication among healthcare providers, RACH residents and family caregivers is essential for safe medication management and meaningful engagement in decisions (Wheeler et al. 2018). Involving residents and caregivers in medication communication improves shared decision‐making, respects preferences and enhances care quality (Naughton 2018; Parker et al. 2021). Person‐centered management integrates residents and caregivers into medication reviews, monitoring, education and administration (Naughton 2018). As residents and caregivers are often the only consistent link across transitions, their engagement is vital for continuity of care and reducing preventable errors (Dowling et al. 2024). However, rural RACH residents face added barriers, including provider shortages and fragmented communication, limiting their involvement in medication management. This study explores the challenges rural RACH residents and their family caregivers face in medication communication during transitions of care. By examining their experiences, the study aims to highlight factors influencing engagement and identify opportunities to improve communication practices, contributing to safer, more person‐centered care in rural settings.

## 3 The Study

### 3.1 Aim

The study aim was to explore how rural RACH residents and their family caregivers experience engagement in medication communication during transitions of care.

The research question was: How do rural RACH residents and their family caregivers navigate and engage in medication communication during transitions of care?

## 4 Methods

### 4.1 Design

This study employed a qualitative exploratory design (Creswell and Creswell 2018) to examine the perceptions and experiences of rural RACH residents and their family caregivers regarding medication communication during transitions of care. This approach was well suited for exploring this under‐researched area, as it enables a deep understanding of complex, context‐specific phenomena (Creswell and Creswell 2018). The design allowed for the capture of detailed insights from those most directly affected, helping to inform targeted interventions that address the unique challenges faced by rural residents and caregivers. The study adhered to the Consolidated Criteria For Reporting Qualitative Research (COREQ) checklist (Tong et al. 2007), ensuring rigour and transparency, with the completed checklist provided in File S1 (the Consolidated Criteria For Reporting Qualitative Research (COREQ) checklist).

### 4.2 Theoretical Framework

The study applied the Patient and Family Engagement Framework (Carman et al. 2013) to assess how rural RACH residents and their family caregivers engage in medication communication during transitions of care. The framework defines engagement across three levels:

- Consultation—passive engagement, where patients and family caregivers primarily receive health information with little input.
- Involvement—represents a more collaborative approach, where patients and family caregivers actively participate in discussions and decision‐making.
- Partnership/Shared Leadership—patients and family caregivers act as equal partners, working with healthcare providers to guide and influence healthcare practices (Carman et al. 2013).

Applying this framework helped categorise engagement levels, highlighting gaps and opportunities to improve medication safety, continuity of care and collaboration in rural settings.

### 4.3 Study Setting and Sampling

This study was conducted in two government‐operated RACHs in rural New South Wales (NSW) and Victoria, Australia. Using the Monash Modified Model (Australian Government Department of Health and Aged Care 2024), both sites are classified as MMM5 (Monash Modified Model Category 5). Category MMM5 represents small rural towns with a population of fewer than 5000 people. These towns are often characterised by significant distance from regional and metropolitan centres, contributing to challenges in accessing healthcare services and resources.

Purposive sampling was applied in this study to ensure that participants met the specific criteria relevant to the research aims (Palinkas et al. 2015). This technique allowed for the selection of individuals who had direct experience or knowledge related to the study's focus, ensuring that data collected would be rich and relevant. Additionally, residents were invited to nominate family caregivers, ensuring that those who played an active role in care were included in the study.

### 4.4 Inclusion and Exclusion Criteria

The inclusion and exclusion criteria used to select rural residential aged care facility sites and study participants are presented in Table 1.

**TABLE 1:** Study inclusion and exclusion criteria.

Criteria	Inclusion	Exclusion
**Rural residential aged care homes**		
Rural residential aged care homes	Located in Victoria or southern NSW to minimise the first author's travel burden during data collection periods	
**Study participants**		
Residents	Permanent residents of rural RACFs Have experienced at least one transition of care in the past 12 months Prescribed at least one medication Can communicate in English Have the cognitive ability to provide informed consent, as assessed by RACH nursing staff	Residents with a life expectancy of less than six months, as assessed by RACH nursing staff. Have significant long‐term impairments (e.g., cognitive, language or motor) that limit their communication abilities and prevent participation independently or with support
Family caregivers	Identify as a current caregiver (e.g., partner, relative or friend) of a resident meeting the above criteria Can communicate in English Can provide informed consent Aged at least 18 years	Have significant long‐term impairments (e.g., cognitive, language or motor) that limit their communication abilities and prevent participation independently or with support

### 4.5 Recruitment Procedures

Outreach to rural RACHs occurred between March and June 2024. Author 1 contacted Executive Directors and Regional Managers via telephone and email to discuss the study and secure support. Invitations were also distributed through rural Primary Health Networks (PHNs) and university newsletters (anonymised for manuscript). Organisations provided written consent for participation. Healthcare providers at participating RACHs identified eligible residents and family caregivers, shared study information, and collected permission‐to‐contact forms, which were then provided to Author 1 during on‐site visits. Interested participants were approached by Author 1 during site visits for residents or via telephone/email for family caregivers to provide study details, address any questions and obtain informed consent. Consent, either written or verbal, was collected before interviews.

### 4.6 Data Collection

Data collection took place between June and July 2024, conducted by Author 1 using a tailored interview schedule and demographic questionnaire. Both the interview guide and questionnaire were specifically developed for this study, drawing on existing demographic questionnaires and interview guides from prior research on transitions of care for older adults (Manias et al. 2015). Participants completed a brief demographic survey (age, gender and education) in hard copy or online via the Research Electronic Data Capture (REDCap) system (Harris et al. 2009). The interview questions included open‐ended questions with prompts as appropriate. For example,

- Residents: What can you tell me about your medications?
- Caregivers: What can you tell me about your loved one's (the resident) medications?
- How important is it for you to communicate about medications during a transition of care?
- What medication information would you like to be communicated during transitions of care?

In‐person interviews with residents were conducted in private areas of the RACH to ensure confidentiality, while family caregivers participated via online interviews. Interviews were recorded using a portable audio recorder for in‐person sessions and Zoom for online interviews. As a token of appreciation, residents received thank‐you cards and small gifts, such as boxes of chocolates. Author 1 had no prior relationship with any study participants.

Information power, a concept used to determine the adequacy of sample size in qualitative research, was applied to the data collection (Malterud et al. 2016). This approach suggests that the more relevant the data to the study aims and the more specific the sample, the fewer participants are required to achieve robust findings. In this study, information power was attained when repeated patterns and themes emerged across participant interview responses during data analysis (Malterud et al. 2016). For example, several participants shared similar experiences regarding challenges in recalling medication details and the need for written information to enhance communication with healthcare providers. As these responses were consistently echoed across multiple interviews, it became evident that sufficient data had been collected to provide a comprehensive understanding of the residents' and family caregivers' perspectives on medication communication during transitions of care. Demographic information was collected from participants through paper‐based questionnaires for in‐person interviews and online questionnaires for web‐based interviews using the secure, REDCap web‐based application designed to support data collection for research studies (Harris et al. 2009). Completed paper questionnaires were manually entered into the REDCap system by Author 1, while online responses were directly captured using REDCap‐designed questionnaires.

### 4.7 Data Analysis

Audio‐recorded interviews were transcribed using Microsoft Word Transcribe, with Author 1 manually reviewing each transcript against the original audio for accuracy and completeness, which was further verified by the second author. Thematic analysis was conducted using both inductive and deductive approaches, following the process outlined by Braun and Clarke (2006). First, Author 1 familiarised themselves with the data by repeatedly reading and re‐reading the interview transcripts to gain an in‐depth understanding. During this stage, initial thoughts and potential patterns were noted. Next, coding was conducted by systematically identifying meaningful sections of the text. These codes were generated directly from the data, ensuring that themes were developed inductively rather than being predetermined. The codes were reviewed, refined and organised into broader groups based on similarities and relationships. NVivo 14 software (QSR International) was used in systematically managing and analysing the data. Once codes were grouped, potential themes were developed by identifying patterns and connections across these groups. These preliminary themes were reviewed to ensure they accurately captured key aspects of participants' experiences. The coding framework was refined iteratively to enhance clarity and consistency. In the final stage, a deductive approach was applied by mapping the emerging themes onto the Patient and Family Engagement Framework (Carman et al. 2013). This framework provided a structured way to classify themes based on levels of engagement in medication communication, from passive consultation to active partnership and shared decision‐making. The second author independently reviewed the transcripts and refined the codes. The two authors then collaborated to examine the themes, ensuring consistency and coherence and discussing any discrepancies or overlaps. This collaborative approach strengthened the rigour of the analysis and helped achieve a comprehensive understanding of participants' experiences with medication management communication during transitions of care. Demographic data were exported from REDCap to a Microsoft Excel spreadsheet and analysed using descriptive statistics, including frequencies, means and ranges, in Excel by Author 1.

### 4.8 Ethical Considerations

This study received approval from the Monash University Human Research Ethics Committee (Project ID: 41401). Formal ethics approval from the overarching aged care organisations was not required; however, they provided written consent for the research to be conducted in their rural RACHs. This consent was necessary for site access and was a requirement of the authors' institutional ethics submission. Participation was voluntary, with written informed consent obtained prior to interviews. Participants could withdraw at any time without affecting their relationship with the RACH or healthcare team. Confidentiality and privacy were maintained, and data will be securely stored for 5 years following the Australian Code for the Responsible Conduct of Research (National Health and Medical Research Council 2018), with access limited to the research team. After 5 years, all data will be destroyed.

To protect vulnerable residents, registered nurses at each RACH site used clinical judgement to exclude those with cognitive or physical limitations. Author 1's experience informed recruitment, with on‐site assessments ensuring participants' wellbeing. Interviews were held in residents' rooms, with access to medical assistance. Family caregivers were identified based on eligibility, contacted with consent, and given 3 days to decide. Interviews were arranged at their convenience, either online or in person. Caregivers were informed that participation was voluntary and withdrawal would not affect their relationship with the RACH or healthcare team.

### 4.9 Rigour and Reflexivity

Author 1 adhered to ethical guidelines, maintained a consistent interview approach, and revisited the research questions to stay focused on the study's aim (Jootun et al. 2009). Reflexivity was practised through journaling after each interview, reflecting on potential biases and their impact on data interpretation (Jootun et al. 2009). Contextual factors, such as geographic location, staffing levels and the RACH environment, were considered as they might influence participants' experiences. For instance, whether staffing limitations or fluctuations could affect medication communication and impact participants' trust and engagement. Differences in educational backgrounds were considered as possible factors affecting their comfort and understanding of medication communication, potentially shaping their participation in medication management decisions. To minimise bias, both authors independently coded the data and collaboratively resolved discrepancies (Creswell and Creswell 2018). This iterative process maintained the integrity of the research.

Both authors are female researchers with extensive experience in qualitative research. Author 1, a Research Fellow (PhD) at Monash University, has 5 years of experience working with older adults and their family caregivers in aged care. Author 2, a Professor (PhD) and Author 1's supervisor, specialises in medication communication and is a qualified pharmacist and registered nurse. Their backgrounds were considered throughout the study, with reflexivity addressing how their roles and expertise could influence the research process and findings.

## 5 Results

### 5.1 Characteristics of the Sample

Of the 15 RACHs initially contacted, five agreed to support the study, but three withdrew before data collection began. The primary reasons for non‐participation were a lack of available staff to assist with resident recruitment (10 RACHs) and insufficient interest in the study (5 RACHs). Ten residents and seven family caregivers declined to participate due to lack of interest, ill health or personal constraints. In addition, two residents withdrew during visits due to sudden health issues, while four caregivers did not respond to follow‐ups.

Sixteen individual interviews were conducted with 9 residents and 7 family caregivers. Two individuals who initially expressed interest in participating later withdrew; one resident due to health issues and a family caregiver who could not be reached despite repeated attempts by Author 1. Refer to Tables 2 and 3 for detailed characteristics of the RACF sites and study participants.

**TABLE 2:** RACH recruitment and characteristics.

	Total number	%
**Recruitment**		
RACH organisations invited to participate in the project	15	
RACH organisations that consented to participate in the project	5	33
Reasons provided by RACH organisations for declining participation		
Insufficient interest in the project topic	4	27
Lack of staff to assist with resident recruitment	6	40
Number of withdrawals	3	60
**Characteristics of particpating rachs**		
RACHs that took part	2	
Location in Australia		
New South Wales (RACH A)	1	50
Victoria (RACH B)	1	50
Funding		
Government operated	2	100
Number of resident beds		
RACH A (low and medium care beds)	84	
RACH B (low and high care beds)	34	

**TABLE 3:** Resident and family caregiver demographic information.

Characteristic	Residents, *n* (%)	Caregivers, *n* (%)
**Participant characteristics**		
Age (years)		
Mean	85 years	62 years
Range	69–96 years	54–71 years
Gender		
Man	3 (33%)	2 (29%)
Woman	6 (67%)	5 (71%)
Country of birth		
Australia	7 (78%)	7 (100%)
Other	2 (22%)	
Primary language		
English	9 (100%)	7 (100%)
Highest education attained		
Primary school	4 (44%)	
High school	4 (44%)	2 (29%)
Diploma/certificate	1 (12%)	1 (14%)
Bachelor's degree		1 (14%)
Master's degree		2 (29%)
PhD		1 (14%)
Time living in RACH (years)		
Average	2.0 years	
Range	0.3–4.5 years	
Current work situation		
Employed		2 (29%)
Semi‐retired		2 (29%)
Retired		2 (29%)
Other		1 (14%)
Location		
Rural Australia	9 (100%)	5 (71%)
Regional Australia		
Metropolitan Australia		1 (14%)
Other		1 (14%)

### 5.2 Themes

The analysis identified two main themes that reflected residents' and caregivers' experiences and perceptions of medication communication during transitions of care, emphasising key aspects of their involvement in the process. Both themes were further divided into two sub‐themes each.

5.2.1 Medication communication during transitions into and between healthcare services:
  5.2.1.1 Medication communication challenges following residents' transitions to an aged care home.
  5.2.1.2 Residents' and caregivers' experiences of medication communication during residents' hospital stays.
5.2.2 Influences shaping residents' and caregivers' engagement in medication communication:
  5.2.2.1 Resident and caregiver knowledge, beliefs and preferences of medications.
  5.2.2.2 External elements influencing residents' and caregivers' engagement in medication communication.

#### 5.2.1 Medication Communication During Transitions Into and Between Healthcare Services

This theme explores the experiences of rural RACH residents and their family caregivers regarding medication communication during transitions, framed through the Patient and Family Engagement Framework. It highlights shifts in their involvement, from active participation (shared decision‐making and partnership) to more passive roles (consultation and passive reception). These shifts led to communication challenges and inconsistencies, with varying levels of engagement for both residents and family caregivers across different healthcare settings.

##### 5.2.1.1 Medication Communication Challenges Following Residents' Transitions to an Aged Care Home

This sub‐theme explores the experiences of residents and family caregivers following their transition from home to a rural RACH, focusing on changes in medication communication. It highlights the shift from active participation, such as shared decision‐making, to more passive roles, where residents and family caregivers received information in a consultation capacity. This shift created challenges in understanding medication changes and reduced their active involvement in care.

Following the transition to a RACH, many residents experienced reduced engagement in medication management, shifting from a more hands‐on role to a consultation role. The following statement from a resident illustrates the shift from independent management to reliance on healthcare staff, marking a loss of control over their medication routine:

I used to handle all my medication myself…but when I came here, they wouldn't allow that. (300)

Despite being excluded from decision‐making, residents maintained control by monitoring their medications, tracking appearance and quantity. One resident shared their attempt to stay informed and involved, even though they had no direct control over medication decisions:

I keep up with what's going on by the number of tablets I take and what they look like. (300)

Family caregivers also experienced a shift from active decision‐making to a more passive consultation role. One caregiver noted the change:

I used to be heavily involved…Now that doesn't happen as much as the nurses do everything. (C206)

While some family caregivers missed being actively involved, others felt relief from the reduced responsibility. One caregiver reflects on the mixed emotional responses to this change, noting that some caregivers appreciate the relief from the pressure of managing medications:

It's a bit of a relief not having to do everything…I don't know that I would be able to cope now with all the medications that my mother is on. (206)

Both residents and family caregivers emphasised the importance of being informed about medication changes, often feeling disengaged due to inconsistent communication. One resident expressed the desire for more transparency:

I want to know what was changed and why. I want to be told as I'm the one putting them (the medications) in my body. (301)

Similarly, a family caregiver voiced the need for clearer explanations:

If changes are made, I would like to know at least why and if there are any side effects. (200)

Although residents expressed a need for information, engagement at the consultation level was often inconsistent, leading to frustration. One resident shared their experience of repeatedly asking about a medication change but receiving unsatisfactory answers, emphasising the frustration of not having their concerns addressed.

I keep asking about the stopping of one of my medications…they just repeat the same thing and never answer my question. (302)

Residents' and family caregivers' engagement often depended on their proactive efforts to seek information. Many residents reported feeling supported when they initiated conversations with RACH nursing staff:

The girls here are very good at explaining if I ask. They will usually sit on my bed and explain everything to me. (303)

Similarly, family caregivers had to request updates from the staff:

(When the RACH nurses contact me)…It's just a general conversation, maybe that a painkiller has been increased…I usually have to ask if I want to know why something was changed because they don't give that information unless I ask. (205)

Limited direct communication with visiting general practitioners (GPs) also created barriers to engagement, as one family caregiver explained:

I've never seen or spoken to the doctor that visits. It's the nurses who tell me if he's changed something. (204)

This lack of direct communication further hindered residents and family caregivers from feeling fully involved in medication decisions. Additionally, some residents felt their medication concerns were often overlooked, as GPs focused on other health issues:

I've asked a few times about my tablets, but he (the GP) just wants to talk about my cancer. (308)

Many residents noticed a decline in communication with GPs after transitioning to aged care, compared to their prior experiences. One resident reflected on the challenges of maintaining the same level of involvement in their care, recalling a more engaging relationship with their previous doctor:

The doctor I had before when I was living at home, was really good. She would tell me which tablet was for what. Kept me involved, you know. The doctor who comes here doesn't talk much unless I start the conversation. (301)

##### 5.2.1.2 Residents' and Caregivers' Experiences of Medication Communication During Residents' Hospital Stays

This sub‐theme explores the communication challenges faced by rural RACH residents and their family caregivers during hospital stays, focusing on varying levels of engagement in medication communication. Residents and family caregivers often encountered fragmented communication, limited involvement in medication discussions, and rushed interactions, particularly in larger hospitals. These experiences reflect a shift from active engagement, such as shared decision‐making, to passive roles, where information was provided without input.

In larger regional and metropolitan hospitals, residents often felt left out of medication discussions, receiving little to no information about changes to their medications. One resident recalled an unsatisfactory interaction with a nurse, highlighting the lack of detailed communication and the impersonal nature of the exchange, which left the resident feeling uninformed and dissatisfied:

I wasn't satisfied with the explanation given to me by the nurse…She just said: It's just what the doctor ordered. (306)

Some residents, especially those who were acutely ill, found it challenging to engage in discussions with hospital providers in larger settings. One resident, who had suffered a stroke, shared their difficulty in recalling any details of the conversation, indicating that health conditions, particularly in acute situations, can impair residents' ability to participate in medication discussions, leaving them feeling disconnected from their care.

I had a stroke and was really crook…I really don't remember if they spoke to me. (307)

Other residents actively sought clarification but remained dependent on hospital healthcare providers for information. One resident expressed their frustration with not understanding medication changes:

I kept saying that I don't understand why my medications have changed and I want you to tell me. (301)

This quote highlights the resident's active involvement in seeking information, but also their reliance on hospital staff to provide the necessary explanations.

Residents sometimes only became aware of medication changes when they noticed physical differences in their medications. One resident's statement highlights the lack of proactive communication from healthcare providers, forcing residents to rely on their own observations to identify changes in their medication:

I said why I had this particular tablet. I said I don't recognise that. (301)

Delays in receiving medications were another common issue in larger hospitals, which further frustrated residents, especially when no explanation was provided. One resident recounted:

For 24 hours I didn't have any medication at all. None at all. They didn't tell me why, even when I kept asking. (305)

Family caregivers often felt left out of the communication process, with many only receiving information when discharge was imminent. One caregiver shared their experience, highlighting how caregivers were frequently excluded from important conversations until it was too late to influence care decisions:

No one spoke to me…Only when (my mother) was ready to leave. (201)

The need to actively chase information added to their frustration:

When I finally got there (to the metropolitan hospital), the decisions had been made, and I had to chase them down for information. (204)

The fast‐paced nature of larger hospitals also made it challenging for caregivers to engage with healthcare providers. One caregiver noted:

They were always really busy, and I felt like I was inconveniencing them. (203)

In contrast, caregivers described communication in smaller rural hospitals as more personalised and attentive. Despite acknowledged challenges with healthcare access in rural areas, these settings were seen to facilitate better engagement by fostering familiarity and stronger relationships with residents and caregivers. For instance, one caregiver noted:

We're spoilt at the (local rural hospital)…at least they take the time to tell you what's happening. (201)

This quote underscores the value of individualised attention and communication in smaller, local hospital settings, where staff took the time to keep caregivers informed.

Familiarity with healthcare providers working in the smaller rural hospitals also improved communication:

The doctor at the (local rural) hospital also works at the (aged care) home so he knows mum pretty well…He doesn't need to ask a lot of questions because he knows mum's medical history from his visits to the home and when she's up at the hospital. That makes it all very easy. (206)

Another family caregiver highlighted the importance of personal connection, contrasting it with the impersonal nature of larger hospitals. They noted that familiarity and a personal rapport can lead to more compassionate and effective communication, something that is often lacking in bigger healthcare settings.

I grew up with some of the nurses who work at the (local rural) hospital, so they know mum and I really well. They come and speak to you, like family really. That doesn't happen in the bigger hospitals where you are just a body in the bed. (204)

#### 5.2.2 Influences Shaping Residents' and Caregivers' Engagement in Medication Communication

This theme explores how residents' and family caregivers' knowledge, beliefs and preferences influence their engagement in medication communication during transitions of care, in line with the Patient and Family Engagement Framework. Limited knowledge or memory challenges led to passive roles, reflecting passive consultation. In contrast, written information and online research fostered active involvement, aligning with shared decision‐making and partnership. Trust in healthcare providers and family members shaped engagement but sometimes caused hesitancy in questioning decisions. External factors, like staffing shortages and cultural differences, also impacted communication, emphasising the need for consistent support and strategies to encourage active participation.

##### 5.2.2.1 Resident and Caregiver Knowledge, Beliefs and Preferences of Medications

This sub‐theme encompasses how residents' and family caregivers' knowledge, beliefs and preferences about medications influenced their engagement in communication. Many felt excluded from discussions, reflecting passive consultation. Others actively sought information through written lists or online research, aligning with shared decision‐making. Trust in healthcare providers or family members often led to passive roles, with hesitancy to question decisions, reflecting lower levels of engagement.

Many residents struggled to remember details about their medications, such as names and purposes, due to the large number they were prescribed. One resident expressed the challenge of managing multiple medications, noting how the sheer volume made it hard to keep track.

I take 21 tablets a day and it's hard to keep track. (300)

Another resident noted a loss of knowledge after transitioning to a RACH, emphasising the passive role they had taken in medication management:

Now that I don't look after them, I've lost all knowledge because no one tells you anything about them unless you ask. (302)

Memory issues, particularly those associated with ageing, compounded the difficulty in keeping track of medications. As one resident shared:

I can't remember them all because there's so many. That's just a part of growing old. (304)

This reflects a common frustration felt by residents, who often found themselves excluded from discussions about their medications. Several residents expressed their desire for more proactive communication, as one shared:

I don't really know much about them now…they never tell me. I would like to be told so I can ask questions. (303)

Family caregivers also encountered challenges, especially after the transition to a RACH. One caregiver shared that the shift of responsibility to institutional care often led to a sense of disengagement, even for caregivers themselves:

I must confess, I haven't taken much notice since mum moved into the home. (205)

Despite these challenges, both residents and caregivers expressed a desire for better knowledge, particularly through written materials. One family caregiver explained:

If I had it written down, it might make me more inclined to talk about meds with the staff. It would give a bit of knowledge, confidence to talk to the doctors and nurses, you know. (204)

A resident echoed this preference for written medication information, stating:

I think it's necessary to have it written…because it's hard to remember, especially when you're taking so many tablets like I am. (300)

This reinforces the value of tangible, written resources in enhancing residents' understanding of their medication regimens. One resident noted:

I can't remember everything they tell me because I take so many. I'd like a list telling me what each tablet is for, something in simple English that I can understand. (303)

Some residents took proactive steps to seek out information online, demonstrating initiative in understanding their medications. One resident shared:

…The first thing I do when I get something new is go into Google and just find out about it (306)

A family caregiver described a similar approach:

If I don't understand something, I will look it up on the internet. It helps me feel more comfortable talking to the staff about mum's medications. (203)

This demonstrates how residents and family caregivers take ownership of their knowledge, despite the challenges.

Residents' and caregivers' trust in healthcare providers, combined with personal experiences, shaped their communication and involvement during care transitions. Many participants trusted healthcare providers to manage medications, which led to more passive involvement. One resident shared:

I leave looking after medications to the doctor. He knows more than me. (303)

This reflects a deferment of responsibility to healthcare providers, as residents trust their expertise. A family caregiver noted their own reliance on healthcare providers:

I am not a doctor or nurse so I can't contribute anything. I just have to trust what they say. (201)

Some residents and caregivers deferred responsibility to family members in medical professions, which further reduced their direct involvement. One family caregiver explained:

One of my sisters is a RN and she takes care of all that. I probably should know more but my sister has all the knowledge about meds so I leave it to her. (200)

Similarly, a resident relied on their son to manage their medications, trusting his younger age and memory:

I leave all that to my son. He has a young mind and can remember it all. There are too many for me now to keep track of. (306)

This reliance on family members reflects a desire for support when navigating complex medication regimens. However, this trust sometimes led to hesitancy in questioning healthcare providers. One family caregiver shared:

I just accept that they know what they are doing. Though sometimes I do wonder if all the medications do what they're supposed to as they are all taken together. But I don't have the expertise to ask. (204)

A resident expressed a similar feeling, acknowledging uncertainty about their medications but suggesting they trust their regimen due to the lack of negative effects:

I am on so many and I don't know why or what they are for. But I am still alive so they must be doing something. (306)

##### 5.2.2.2 External Elements Influencing Residents' and Caregivers' Engagement in Medication Communication

This sub‐theme captures how systemic and contextual factors impact residents' and caregivers' engagement in medication communication. In rural settings, issues like staffing shortages, high turnover and language/cultural differences hindered meaningful communication and participation, limiting opportunities for active involvement in decision‐making.

A key challenge in rural areas was the high turnover of healthcare providers, which led to fragmented care and disrupted communication. This instability made it difficult to establish trust with healthcare providers, affecting residents' and caregivers' involvement in medication decisions. A family caregiver explained the delays caused by inconsistent provider access:

It (the changes to mother's medications) was a bit slow in some cases because the Flying Doctor couldn't come in and sign them off. (206)

This quote reflects how the reliance on transient healthcare workers hindered timely medication management and contributed to delays in care.

Residents also expressed anxiety about unfamiliar staff, particularly when it came to administering medications. One resident recounted a close call with incorrect medication:

One day, one of them (agency nurses) came in to give me my tablets. When she handed them to me, I noticed they didn't look like my tablets. I said to her, ‘What are you giving me? These aren't mine.’ She checked the list and said, ‘Oh, sorry, these aren't yours.’ That gave me a scare because if I'd taken them without checking, who knows what might have happened? (306)

This highlights the fear residents have when they encounter unfamiliar staff, especially when medication safety is at stake. It also shows the heightened vigilance some residents develop to protect themselves in such situations.

Family caregivers also expressed concerns about the frequent rotation of staff and its potential for errors. One noted:

There seems to be different ones (healthcare providers) every day. I'm surprised there's not more problems as bringing in new staff all the time is just asking for mistakes. Unfortunately people don't want to stay because (the rural town) is so far from anywhere. (206)

This reflects the difficulty of maintaining continuity of care in rural areas, where geographic isolation leads to staffing shortages and turnover, which can negatively impact care quality.

The frequent rotation of staff caused frustration for both residents and caregivers, who had to repeatedly share medical histories and medication concerns with new providers. One family caregiver expressed how the lack of continuity in rural healthcare creates frustration, as constantly repeating important details hampers progress and fosters a sense of instability in care.

It's been hard to get any traction, you know, because (the rural town) has been short of healthcare workers…It gets so frustrating, having to repeat things over and over because the ‘blow‐ins’, as I call them, don't know anything about the people they treat. It's so much better when there's a full‐time doctor in town, because they work at the hospital and nursing home. They get to know the residents really well. (206)

Language barriers were also a significant issue, with residents and caregivers struggling to understand staff from overseas. One resident shared:

A lot of them come from overseas because they can't get anyone else to come out here. Some of them are hard to understand. I don't understand them and they don't understand me. I gave up trying to talk to them. So now, if I want to know something, I wait for one of the other nurses to come on. (307)

Family caregivers also expressed similar concerns, citing how language barriers affected their ability to communicate effectively with staff:

Because the home is so isolated, they can only get staff from countries like the Philippines or India to work there. You can't understand some of them. It can put you off talking to them, really. (205)

These statements highlight how language differences can lead to communication breakdowns, resulting in disengagement from both residents and caregivers. They emphasise the challenges of rural, isolated settings, where reliance on overseas staff can create barriers that hinder effective engagement between residents, caregivers and healthcare providers.

## 6 Discussion

This study is the first known Australian investigation into the experiences of rural RACH residents and their family caregivers regarding medication communication during transitions of care in rural settings. The findings reveal significant challenges faced by residents and caregivers that have not been widely reported before. These challenges include the influence of healthcare provider turnover and reliance on transient staff, which intensify communication difficulties in rural RACHs, as well as concerns about communication breakdowns with international healthcare providers, particularly those from culturally and linguistically diverse (CALD) backgrounds. The study also highlights subtle but important underlying factors such as power imbalances and ageism that influence how residents and caregivers engage in medication discussions, along with the consequences of transitioning to a rural RACH, particularly the loss of autonomy over medication management. Overall, the findings indicate that most rural RACH residents and family caregivers primarily engage at the consultation level, where they are informed of medication changes but have limited influence over decisions. These findings emphasise the need for clear, consistent communication during transitions of care, especially in rural areas where staffing shortages and high turnover are prevalent.

This study highlights the impact of healthcare provider turnover and transient staff on medication communication in rural RACHs during transitions of care. These challenges hindered residents and family caregivers from engaging in medication discussions, reducing communication quality. Turnover and reliance on transient staff disrupted continuity of care, as new providers lacked familiarity with residents' histories, preferences and medication regimens (Shen et al. 2023; Ma et al. 2022). Rural areas in Australia face healthcare workforce shortages, with provider numbers nearly 60% lower than urban settings (Blackberry and Morris 2023). These shortages are expected to worsen due to limited resources and an aging population (Blackberry and Morris 2023; Wilkes 2025). This makes effective medication communication even more critical. Provider turnover and shortages can hinder trust, which is essential for safe medication management and effective communication (Thwaites et al. 2023; Yang et al. 2021). Our study shows that frequent healthcare provider changes prevent residents and family caregivers from building stable, trusting relationships, which are vital for clear medication discussions (Lynch et al. 2022; Stephen 2024). This instability can lead to inconsistent care, where medication changes may not be communicated effectively, and residents' preferences are overlooked (Dowling et al. 2024; Krein et al. 2022; Manias et al. 2020). Limited provider supply also affects collaborative, patient‐centred care (Coombs et al. 2022). Residents and caregivers may feel less empowered to engage in medication discussions, especially if healthcare providers are transient or unfamiliar with individual needs (Damiaens et al. 2022; Shen et al. 2023). Structuring medication communication to actively involve residents and family caregivers could help mitigate the impacts of workforce challenges.

Participants in our study identified a key consequence of transitioning to a RACH: the loss of autonomy over medication management. Many residents and family caregivers reported feeling disempowered and frustrated, as they relinquished control over medication decisions. This loss of agency created barriers to communication, with residents and family caregivers feeling excluded or hesitant to raise concerns. Previous research has examined the emotional impact of RACH transitions, like loss of independence (Johnson and Bibbo 2014; Lee et al. 2002) and loneliness (Koppitz et al. 2017; St Clair et al. 2022), but little has focused on medication communication. Our study, along with prior research, highlights that many residents and family caregivers want to remain involved in medication discussions (Dowling et al. 2024). Understanding how relocation affects medication communication is crucial for improving care quality and ensuring active involvement, especially in rural areas with heightened healthcare challenges (Piper et al. 2015). Our findings suggest limited engagement in medication discussions can lead to knowledge gaps, increased dependency and uncertainty about treatment. Loss of autonomy has been linked to depression (Dow et al. 2011; Tan et al. 2023) and lower well‐being (Ratnakaran 2023), with rural communities being more vulnerable due to restricted access to specialised services (National Rural Health Alliance 2022). RACH providers should prioritise transparent communication needs, involving residents and family caregivers in decisions to improve care and medication safety.

Participants also noted that verbal communication with healthcare providers from international or culturally and linguistically diverse (CALD) backgrounds could be challenging due to differences in accents or speech patterns. Some residents and family caregivers had difficulty in understanding these providers, which they felt affected medication discussions and limited their involvement in decision‐making. Clear communication is essential for safe medication management (Squires 2017), and these barriers are particularly significant in aged care, where residents may face hearing loss, cognitive impairment and difficulties with medical terminology (Gu and Shah 2019). These issues may be more pronounced in rural Australian communities, where multiculturalism is still emerging, and exposure to diverse communication styles is limited (Wang et al. 2023). Australia's increasing reliance on internationally trained healthcare providers to address rural workforce shortages emphasises the need to address these communication challenges (Murray and Craig 2023). While language barriers in CALD patients have been studied (Gu and Shah 2019), the impact of healthcare providers' speech patterns on engagement in rural healthcare settings remains underexplored (Murray and Craig 2023). Previous research links communication barriers to poorer health outcomes, increased healthcare use and adverse events (Divi et al. 2007; Squires 2017). Given the multiple communication challenges already faced in rural RACHs, this study highlights the need for strategies to support clear communication, such as raising awareness among international providers about the importance of clarity and simplicity.

This study found that power imbalances and ageism may subtly influence how rural RACH residents and their family caregivers experienced and navigated medication communication during transitions of care. These factors were identified as explicit themes but emerged as underlying dynamics woven throughout participant accounts. Many participants described deferring to healthcare providers, particularly doctors, viewing medication decisions as ‘their job’ or assuming providers inherently knew best. This reflected ingrained hierarchical relationships that often left residents and caregivers hesitant to question or actively participate in medication decisions (Dowling et al. 2024; Ozavci et al. 2022). While not overtly stated by participants, these power differentials were evident in their limited involvement and perceived marginalisation in medication discussions, aligning with prior findings that residents and family caregivers frequently occupy passive roles (Dowling et al. 2024). Such dynamics stem from authority structures, communication styles, cultural norms and organisational factors (Ocloo et al. 2020) and appeared intensified in rural settings by high staff turnover and resource shortages disrupting trust and continuity of care (Jones et al. 2021). Ageism may also subtly complicate communication experiences. Residents often linked their difficulties understanding medication information to their age and described receiving limited or unclear explanations. This likely reflects implicit biases and paternalistic communication patterns that diminish residents' empowerment and engagement (Araújo et al. 2023; Ouchida and Lachs 2015). Participants noted variability in memory for medical information depending on how it was delivered, highlighting the need for clear, repeated and multimodal strategies such as teach‐back techniques to support understanding (Latorre‐Postigo et al. 2017; Levy and Macdonald 2016). Although these challenges were underlying rather than explicitly highlighted, they had tangible effects on engagement. Some participants described using adaptive approaches, such as asking clarifying questions or involving family advocates, to navigate power dynamics and enhance their participation. However, workforce shortages and frequent staff turnover in rural aged care limited opportunities for personalised communication and relationship‐building, further constraining engagement.

This study applied the Patient and Family Engagement Framework (Carman et al. 2013), to explore engagement in medication communication among rural RACH residents, family caregivers and healthcare providers during transitions of care. According to this framework, engagement exists on a spectrum, ranging from consultation (passive) to partnership and shared leadership (active) (Carman et al. 2013). Our findings show that while many residents and family caregivers made efforts to engage in medication communication, their involvement often remained at the consultation level, receiving information about medication changes with limited input or influence on decision‐making. These findings are consistent with those from systematic reviews of non‐rural RACH settings (Dowling et al. 2024; Manias et al. 2020). However, this study uniquely examines rural RACH residents, a population that has been underexplored in prior research. Existing research, which applied the framework to non‐RACH patients and family caregivers in various healthcare contexts, shows that higher engagement improves health outcomes, satisfaction and self‐management (Boivin et al. 2018; Hibbard and Greene 2013). These findings highlight the need to engage RACH residents and their caregivers in meaningful discussions to empower them in decision‐making. The findings of this study highlight the importance of strategies to move beyond consultation, fostering active involvement and improving both care experiences and health outcomes.

### 6.1 Strength and Limitations of the Work

The study has notable strengths. It is one of the first to apply the Patient and Family Engagement Framework (Carman et al. 2013) to rural RACHs, providing a structured assessment of engagement in medication discussions. By including residents and family caregivers, it provides a comprehensive understanding of communication challenges and facilitators. The study highlights key rural healthcare challenges, including high provider turnover, reliance on transient staff, and communication barriers with international providers from CALD backgrounds. It also explores the unique difficulties faced by rural RACH residents and family caregivers, such as geographic isolation and limited healthcare access. By applying the framework, the study identifies engagement levels and underscores the need for stronger resident and family caregiver involvement in medication decisions, offering valuable insights to improve care in rural RACH settings.

However, the study has limitations. Recruitment was limited to two rural RACHs, and expansion was limited by staffing shortages and COVID‐19 outbreaks. The research team's geographical distance required reliance on RACH staff for recruitment and material distribution, which may have led to inconsistencies. Scheduling interviews in blocks may have further restricted participation, limiting diversity. The rural focus may reduce transferability to urban or international settings, and the sample may not fully represent the diversity of rural experiences. While the qualitative approach provides rich insights, it may not apply universally across aged care contexts. Despite these limitations, the study generated meaningful insights into resident and caregiver engagement in medication management.

### 6.2 Recommendations for Further Research

Future research should focus on improving medication communication in rural RACHs, addressing workforce shortages, provider turnover, and continuity of care through structured protocols and digital tools. Studies should explore ways to enhance resident and family caregiver involvement in decision‐making, including co‐designed engagement strategies and tailored educational materials. Investigating communication barriers with international providers is also an area worth further exploration, with a focus on training in cultural competency, plain language use and accessibility for residents with hearing or cognitive impairments. Lastly, research should include healthcare provider perspectives to identify system‐level challenges and inform policies that support resident‐family‐centred care.

### 6.3 Implication for Policy and Practice

This study highlights the need for improved medication communication during transitions of care into rural RACHs. A practical approach involves asking residents and family caregivers about their preferences for receiving medication updates and ensuring these are communicated to relevant healthcare providers, including visiting doctors. While Australian RACHs must notify nominated next of kin of significant medication changes (Department of Health and Aged Care 2024), they could also inform non‐nominated family caregivers upon request while clarifying their specific obligations regarding information‐sharing.

Proactive strategies like scheduled meetings and written medication summaries can also improve communication. Staff training in empathetic communication and shared decision‐making is vital. Rural RACHs or their governing bodies should provide medication education and clear communication pathways. Strategies to address communication barriers could include training healthcare providers in plain speech, adaptive language and verification of understanding through teach‐back methods, where residents and caregivers repeat key information, which can support comprehension (Yen and Leasure 2019). Professional development in communication skills, cultural awareness and visual aids can further improve engagement. Digital application tools like MedAdvisor, Healthdirect and MedicineWise can aid medication management and reduce errors. This study also highlights the need to address subtle but important underlying factors such as power imbalances and ageism that influence how residents and caregivers engage in medication discussions. Communication approaches that promote resident and caregiver empowerment, such as multimodal strategies (e.g., combining verbal explanations with written summaries and visual aids) can enhance understanding and participation, especially when frequent staff turnover limits relationship‐building.

Communication breakdowns during transitions to hospitals remain a challenge. Using standardised tools like Identify, Situation, Background, Assessment and Recommendation (ISBAR) (Burgess et al. 2020) and TeamSTEPPS (McCaskill 2011) can improve structured communication. Ensuring clear, accessible transition documentation is crucial, as existing RACH transition documents are often redundant or inaccessible in hospital settings, such as emergency departments, reducing their utility for hospital staff (McCloskey 2011).

## 7 Conclusion

This study underscores the importance of improving medication communication for rural RACH residents and their caregivers, particularly during transitions of care. The findings highlight that the current level of engagement is often limited to consultation, with residents and caregivers having minimal influence on medication decisions. This research highlights the need to move beyond merely informing residents and caregivers, by actively involving them in medication discussions to build trust and shared understanding. For example, asking how they prefer to receive updates and ensuring this is communicated across care teams can promote a more inclusive approach. Addressing communication barriers, particularly in rural areas with workforce shortages and high staff turnover, is critical for improving care experiences. Future studies should focus on developing and evaluating interventions that promote collaboration between residents, caregivers and healthcare providers, ultimately empowering individuals to make informed decisions about their care.

## Author Contributions

The research study was conceptualised by A.D. and E.M. Data curation was performed by A.D. Formal analysis was carried out by A.D. and E.M. Both A.D. and E.M. were involved in data analysis and interpretation. A.D. wrote the original draft, while E.M. reviewed and edited it. All authors have approved the final version of the review.

## Ethics Statement

This study adhered to the ethical principles outlined in the Declaration of Helsinki (49) and received approval from the Monash University Human Research Ethics Committee (Project ID: 41401). Participation was voluntary, with written consent obtained before interviews. Participants could withdraw at any time, and confidentiality was maintained. Data will be securely stored for 5 years, with electronic data password‐protected and hard copies stored in locked cabinets. After 5 years, data will be destroyed, and identifiers will be kept separate from de‐identified responses.

## Conflicts of Interest

The authors declare no conflicts of interest.

## Supporting information


Appendix S1.

## Data Availability

Research data are not shared.
