# Supplementary material for: Engagement in Medication Communication During Transitions of Care for Rural Aged Care Residents and Family Caregivers: A Qualitative Study
Source: J Clin Nurs. 2025 Jul 25;35(2):850–65. doi: 10.1111/jocn.70047 (PMC12779257; doi:10.1111/jocn.70047)
Supplement: Supplementary file 1 — Appendix S1. [file JOCN-35-850-s001.docx]

**Supplementary file 1**

Consolidated criteria for reporting qualitative studies (COREQ): 32-item checklist

| **No** | **Item** | **Guide questions/description** | **Page no in manuscript/comment** |
| --- | --- | --- | --- |
| **Domain 1: Research team and reflexivity** |  |  |  |
| Personal Characteristics |  |  |  |
| 1. | Interviewer/facilitator | Which author/s conducted the interview or focus group? | p9 (4.6 Data Collection) |
| 2. | Credentials | What were the researcher's credentials? *E.g. PhD, MD* | p14 (Rigour and reflexivity) |
| 3. | Occupation | What was their occupation at the time of the study? | p14 (Rigour and reflexivity) |
| 4. | Gender | Was the researcher male or female? | p14 (Rigour and reflexivity) |
| 5. | Experience and training | What experience or training did the researcher have? | p14 (Rigour and reflexivity) |
| Relationship with participants |  |  |  |
| 6. | Relationship established | Was a relationship established prior to study commencement? | p6 (4.6 Data Collection) |
| 7. | Participant knowledge of the interviewer | What did the participants know about the researcher? e*.g. personal goals, reasons for doing the research* | The participants did not know any of the researchers. However, all participants knew that the interview was for research purposes. |
| 8. | Interviewer characteristics | What characteristics were reported about the interviewer/facilitator? e.g. *Bias, assumptions, reasons and interests in the research topic* | None.Participants were introduced to the research using the ethics approved Explanatory Statement and Consent Form. |
| **Domain 2: study design** |  |  |  |
| Theoretical framework |  |  |  |
| 9. | Methodological orientation and Theory | Exploratory descriptive qualitative approach was used to gather in-depth insights into the topic of interest (medication management communication during transitions of care) by exploring it from the perspective of participants, without the constraint of predefined hypotheses. In this type of study, the goal was to explore and describe the experiences and perceptions of participants. Thematic analysis was used to analyze the study data. | p1, (Design)  p6; 4.1 (Study Design) |
| Participant selection |  |  |  |
| 10. | Sampling | How were participants selected? *e.g. purposive, convenience, consecutive, snowball* | P8 (4.2 Study setting and sampling) |
| 11. | Method of approach | How were participants approached? e*.g. face-to-face, telephone, mail, email* | Various methods of approach were used. These are explained under Section 4.5 Recruitment (p8) |
| 12. | Sample size | How many participants were in the study? | p1 (Results)  p15 (5. Results) |
| 13. | Non-participation | How many people refused to participate or dropped out? Reasons? | P15 (5. Results) |
| Setting |  |  |  |
| 14. | Setting of data collection | Where was the data collected? e*.g. home, clinic, workplace* | Reported under 4.2 Study setting and sampling (4.2; p8) |
| 15. | Presence of non-participants | Was anyone else present besides the participants and researchers? | Nil, not relevant. |
| 16. | Description of sample | What are the important characteristics of the sample? *e.g. demographic data, date* | Described in Tables 2 & 3 (RACH and Participant Characteristics) |
| Data collection |  |  |  |
| 17. | Interview guide | Were questions, prompts, guides provided by the authors? Was it pilot tested? | Interview guide and demographic questionnaire were developed and used. These are explained under ‘Data collection’ (4.6; p5-9) |
| 18. | Repeat interviews | Were repeat interviews carried out? If yes, how many? | No repeat interviews were carried out as this was not relevant to the study aims or study rationale |
| 19. | Audio/visual recording | Did the research use audio or visual recording to collect the data? | Yes, noted on p10 (Data Collection) and p11 (Data Analysis) |
| 20. | Field notes | Were field notes made during and/or after the interview or focus group? | Journaling was conducted by Author 1 after the interviews (2.9 Rigour and reflexivity’ p13) |
| 21. | Duration | What was the duration of the interviews? | Duration of interviews is provided in Table 3 |
| 22. | Data saturation | Was data saturation discussed? | Data collection; p10. Information power, a concept used to determine the adequacy of sample size in qualitative research, was applied to the data collection |
| 23. | Transcripts returned | Were transcripts returned to participants for comment and/or correction? | No member checks were carried out as this was not relevant to the study aims or rationale for the study. Nor was it practical. |
| **Domain 3: analysis and findings** |  |  |  |
| Data analysis |  |  |  |
| 24. | Number of data coders | How many data coders coded the data? | p8 (2.9 Rigour and reflexivity) and p6 (4.7 Data Analysis) |
| 25. | Description of the coding tree | Did authors provide a description of the coding tree? | Not applicable. |
| 26. | Derivation of themes | Were themes identified in advance or derived from the data? | Themes were derived from the data (p11) inductively and deductively |
| 27. | Software | What software, if applicable, was used to manage the data? | NVivo software was used (p11; Data Analysis) |
| 28. | Participant checking | Did participants provide feedback on the findings? | Not applicable. |
| Reporting |  |  |  |
| 29. | Quotations presented | Were participant quotations presented to illustrate the themes / findings? Was each quotation identified? e*.g. participant number* | Quotations provided under Results section. |
| 30. | Data and findings consistent | Was there consistency between the data presented and the findings? | Yes, quotes were provided verbatim. Quotations provided under Results section |
| 31. | Clarity of major themes | Were major themes clearly presented in the findings? | Yes (p15-31) |
| 32. | Clarity of minor themes | Is there a description of diverse cases or discussion of minor themes? | Minor themes provided in Results section (p15-31) |
